# Roadmap for the development of alternative test methods

**DOI:** 10.1007/s00204-020-02888-y

**Published:** 2020-08-28

**Authors:** Tim Brecklinghaus

**Affiliations:** Leibniz Research Centre for Working Environment and Human Factors, Ardeystr 67, 44139 Dortmund, Germany

Recently, a roadmap on how to develop in vitro and in silico alternative methods to animal experiments has been published (Sachinidis et al. 2019). The authors suggest a strategy with five milestones:

- Testing of gold standard compounds and pathway controls (Sachinidis et al. 2019; Hengstler et al. 2014). Gold standard compounds are substances for which human toxicity is well understood. Moreover, it should be well established at which concentration ranges a gold standard control should lead to positive and negative results. For example, acetaminophen (APAP) should lead to positive test results at concentrations of 1–2 mM, while concentrations below 0.1 mM should test negative in test methods for hepatotoxicity (Godoy et al. 2013). Pathway controls specifically interfere with specific signal transduction mechanisms. For example, Wnt inhibitors should cause positive test results in test systems of developmental toxicity. Many novel test methods fail already when challenged with gold standard compounds and pathway controls.
- Establishment of test performance metrics. This step requires about 20 positive and negative test compounds of which the blood concentration, e.g., the C_max_ resulting from a specific dosing schedule is known. Moreover, knowledge is required on whether this dosing schedule causes toxicity (positive control) or not (negative control) to a specific organ (e.g., liver or kidney). Based on the lowest positively tested in vitro concentration, two performance indices can be calculated: the toxicity separation index (TSI) informs how well a test system differentiates between toxic and non-toxic compounds; the toxicity estimation index (TEI) is a measure of how well the in vitro test estimates toxic blood concentrations (only for positive controls).
- Iterative cycles of optimization and confirmation. Many test parameters require optimization. A typical example is the exposure period with test compounds. Often the question arises, whether inclusion of an additional readout into the test battery improves performance. This can be objectified by the above-introduced performance metrics TSI and TEI. However, it is important to confirm improvements for independent sets of positive and negative controls to avoid overfitting to a single set of data.
- Evaluation of inter-laboratory reproducibility. This step requires testing of the same compounds by independent laboratories to determine inter-laboratory reproducibility.
- Acceptance by the scientific community and regulatory bodies. Often regulatory bodies have been asked for acceptance of test methods that are by far not sufficiently advanced. Data of at least 300 positive and negative control compounds should be available and conventional performance measures such as sensitivity or specificity should have been determined by independent laboratories.

In recent years, numerous in vitro tests have been developed, particularly for nephrotoxicity (Lee et al. 2017; Jiang et al. 2018; Sjögren et al. 2018), neurotoxicity (Colaianna et al. 2017; Sisnaike et al. 2014; Reffatto et al. 2018; Yang et al. 2018; Keil et al. 2018), developmental toxicity (Adam et al. 2019; Krug et al. 2013; Shinde et al. 2017; Rempel et al. 2015; Waldmann et al. 2014) and hepatotoxicity (Godoy et al. 2013, 2015; Grinberg et al. 2014, 2018; Reif et al. 2015; Leist et al. 2017), often supported by methods of systems modeling (Hoehme et al. 2010; Ghallab et al. 2016; Vartak et al. 2016; Jansen et al. 2017). However, relatively little has been done to quantitatively evaluate how well in vitro/in silico methods resemble the in vivo situation. For this purpose, the road map presented by Sachinidis and colleagues represents a helpful and practical guideline.
